# Large, Omega-3 Rich, Pelagic Diatoms under Arctic Sea Ice: Sources and Implications for Food Webs

**DOI:** 10.1371/journal.pone.0114070

**Published:** 2014-12-04

**Authors:** Steven W. Duerksen, Gregory W. Thiemann, Suzanne M. Budge, Michel Poulin, Andrea Niemi, Christine Michel

**Affiliations:** 1 Department of Biology, York University, Toronto, Ontario, Canada; 2 Department of Process Engineering and Applied Science, Dalhousie University, Halifax, Nova Scotia, Canada; 3 Canadian Museum of Nature, Ottawa, Ontario, Canada; 4 Fisheries and Oceans Canada, Winnipeg, Manitoba, Canada; University of Ottawa, Canada

## Abstract

Pelagic primary production in Arctic seas has traditionally been viewed as biologically insignificant until after the ice breakup. There is growing evidence however, that under-ice blooms of pelagic phytoplankton may be a recurrent occurrence. During the springs of 2011 and 2012, we found substantial numbers (201–5713 cells m^−3^) of the large centric diatom (diameter >250 µm) *Coscinodiscus centralis* under the sea ice in the Canadian Arctic Archipelago near Resolute Bay, Nunavut. The highest numbers of these pelagic diatoms were observed in Barrow Strait. Spatial patterns of fatty acid profiles and stable isotopes indicated two source populations for *C. centralis*: a western origin with low light conditions and high nutrients, and a northern origin with lower nutrient levels and higher irradiances. Fatty acid analysis revealed that pelagic diatoms had significantly higher levels of polyunsaturated fatty acids (mean ± SD: 50.3±8.9%) compared to ice-associated producers (30.6±10.3%) in our study area. In particular, *C. centralis* had significantly greater proportions of the long chain omega-3 fatty acid, eicosapentaenoic acid (EPA), than ice algae (24.4±5.1% *versus* 13.7±5.1%, respectively). Thus, *C. centralis* represented a significantly higher quality food source for local herbivores than ice algae, although feeding experiments did not show clear evidence of copepod grazing on *C. centralis*. Our results suggest that *C. centralis* are able to initiate growth under pack ice in this area and provide further evidence that biological productivity in ice-covered seas may be substantially higher than previously recognized.

## Introduction

Arctic marine food webs are classically viewed as being supported by two temporally and ecologically distinct types of primary production: ice-associated algae and pelagic open water phytoplankton [1]–[3]. It has generally been accepted that under light-limiting ice/snow cover, the water column is unable to support significant numbers of pelagic autotrophs; therefore, ice algae represent the first available food source for zooplankton grazers in the early spring [4]–[6]. Specifically, ice algae have been viewed as the first significant pulse of polyunsaturated fatty acids (PUFA), upon which reproducing zooplankton are reliant. Higher PUFA availability, specifically the long chain omega-3 fatty acid eicosapentaenoic acid (EPA; 20∶5n–3), increases zooplankton egg production and subsequent viability [6], [7]. The timing of maximum ice algae PUFA availability is therefore important to primary consumers and early melting of sea ice could cause a mismatch between food availability and copepod spring hatch.

Contrary to the classical view, increasing evidence suggests that pelagic blooms occur under sea ice throughout the Arctic, sometimes hundreds of kilometers from open water [8], [9]. The dynamics of under-ice pelagic production are poorly understood in comparison to the intense blooms of *Chaetoceros* spp. and *Thalassiosira* spp. that follow ice melt/breakup [8], . Light levels are limited by snow and ice cover, and stratification that concentrates cells in the upper euphotic zone, where conditions are favorable for growth, have not yet formed [8]–[10]. However, failing to account for the contribution of under-ice pelagic production underestimates the net annual primary production of the Chukchi Sea continental shelf by an order of magnitude [11]. Pelagic growth under sea ice may therefore be a significant, yet unaccounted source of PUFA for marine systems and could help buffer food webs from PUFA shortages in years of reduced ice-associated production.

*Coscinodiscus centralis* Ehrenberg is a large centric diatom with a mean cell size of 180–200 µm in diameter, although cell size ranges from 100 to 370 µm in diameter [12]–[14]. The cells are free-living and contain very high amounts of chlorophyll per cell [15]. The distribution of *C. centralis* is reported as cosmopolitan, found in every marine biogeographic region [14], [16]. Reports of its occurrence in Arctic regions date back to the late 1800s [17] and it is a dominant species early in the spring bloom of the North Water Polynya [18]. Although *C. centralis* has been found under the ice at the North Pole in the late summer and early fall [19], most reports are from ice-free areas or marginal ice zones [18], [20]. Cells of *Coscinodiscus* are often found at the base of the euphotic zone, and there is evidence to suggest that they are adapted to very low light irradiances [21]. Despite being present throughout the world's oceans, *C. centralis* is poorly studied and little is known about its growth requirements or general population dynamics [12], [21].

Most observations of phytoplankton growth under ice have been of assemblages dominated by *Chaetoceros* spp. or *Thalassiosira* spp. and were linked to increasing light levels due to melt ponds [9], [22]. We report, for the first time, significant numbers of *C. centralis* under sea ice in the Canadian High Arctic before the onset of melt, challenging our current understanding of Arctic marine food web dynamics. We examined the environmental conditions associated with the presence of *C. centralis* and used fatty acid and stable isotope analyses to provide insights into:

1. The origins of these large-celled diatoms.
2. The potential for *C. centralis* to sustain growth under sea ice.
3. The role of *C. centralis* as a food source for pelagic zooplankton and their potential impact on Arctic food webs.

## Methods

The permit for this study was received from the Nunavut Impact Review Board (NRIB). We confirm that the study did not involve any endangered or protected species. This study did not involve vertebrates. Maps for this manuscript were generated using Ocean Data View v 4.6.1 [23].

### Study area

**Figure 1:**
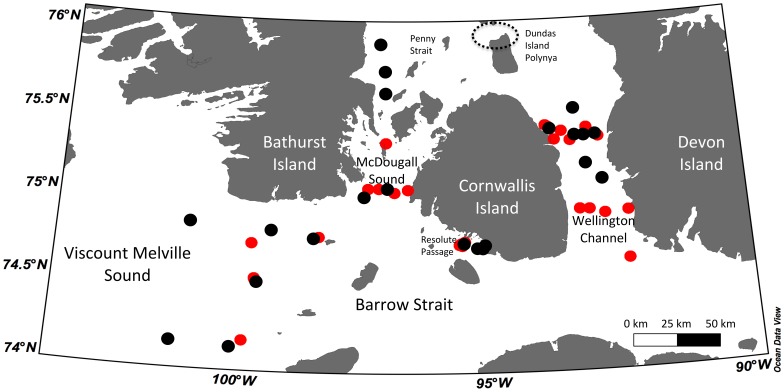
Stations sampled for ice algae and pelagic diatoms during May 2011 (black) and May 2012 (red) around Cornwallis Island, Nunavut. The dotted circle represents Dundas Island Polynya.

Sampling was conducted in a region of first-year sea ice in the Canadian High Arctic, Nunavut, Canada. A total of 47 stations were visited between 1–18 May, corresponding to the ice algal bloom period [4], [22], in 2011 (23 stations) and 2012 (24 stations) (Fig. 1). The main inflows into the study area are from Viscount Melville Sound in the west and Penny Strait to the north with downstream flows through McDougall Strait and Wellington Channel [24]. The general current direction in Barrow Strait is from west to east and averages 6.2 cm s^−1^ in the spring with speeds increasing up to 60 cm s^−1^ during the tidal flux [24], [25]. Southward flow through Penny Strait is about half of the yearly volume compared to the flow through Barrow Strait. With a sill of ca. 125 m, Barrow Strait is the narrowest and shallowest point of the Northwest Passage [10].

### Field observations

Snow depth and ice thickness were measured at each station. Measurements of photosynthetically active radiation (PAR, 400–700 nm) were obtained under the sea ice using a LICOR Li-192S underwater quantum sensor held at the ice-water interface 1m from the hole using a horizontal under-ice arm. All PAR readings were conducted under undisturbed snow cover.

### Sample collection

Bottom ice and pelagic samples were collected at each station. Sea-ice samples were collected using a manual ice corer (Mark II coring system, 9 cm internal diameter, Kovacs Enterprises). The bottom 3 cm of each core was cut and put in a sterile bag (Whirl-Pak, Nasco) and melted overnight with the addition of 500 ml filtered seawater (FSW, 0.2 µm Millipore micropore membrane) to avoid osmotic stress [26]. Ice samples for fatty acid analysis were filtered onto pre-combusted (500°C for 4 h) Whatman GF/F 47 mm glass fiber filters and treated with 10 ml boiling FSW to deactivate lipolytic enzymes [27]. Filters were then stored in cryovials at −80°C. Ice algae collected for stable isotope analysis were filtered on pre-combusted GF/F 21 mm filters and stored in cryovials at −80°C. Samples of *C. centralis* were collected using a zooplankton net (25 cm mouth diameter, 153 µm mesh size) fitted with a flowmeter (General Oceanics model 2030RC) to allow calculation of volume sampled. Three vertical hauls, from 5 m above bottom to the surface, up to a maximum depth of 150 m, were obtained at each station and pooled. Material was then separated by size using 500 µm and 250 µm sieves. Cells of *C. centralis* were isolated from the 250 µm size fraction by letting 500 ml of sample settle for 25 min. This procedure was repeated three times and any remaining zooplankters were removed by handpicking using a Pasteur pipette and a dissecting microscope. These samples, composed predominately of *C. centralis* cells, were then filtered onto pre-combusted Whatman GF/F filters, which were immediately placed into chloroform (Omnisolv grade, VWR) and stored at −20°C until analysis. Isolated samples of *C. centralis* could not be obtained for fatty acid analysis in 2011; rather, qualitative estimates of abundance were made as explained below.

### Cell abundances of *C. centralis*

Qualitative abundance estimates of *C. centralis* in 2011 were made on a 0–3 scale. A value of 3 characterized a sample that, if shaken in a scintillation vial, was completely opaque, a value of 2 indicated a medium level of opacity when the vial was shaken (i.e. other side of vial clearly visible), a value of 1 meant that few specimens were present, and 0 indicated no diatoms were present. In 2012 quantitative subsamples were taken from each site, preserved in Lugol's solution, and counted under a dissecting microscope.

### Feeding experiment

To test whether *C. centralis* could be a suitable food source for copepods, zooplankton, *C. centralis* and ice algae were collected at a single first-year sea ice station in Resolute Passage on 14 May 2011. Calanoid copepods larger than 500 µm in length were retained from pooled vertical hauls taken from 30 m depth to the surface. The copepods were placed in 12 experimental enclosures (500 ml Nalgene polycarbonate bottles) containing 450 ml of FSW, which were placed in a seawater bath at 0°C with light levels of 2.5–3.5 µE m^−2^ s^−1^ during 24 h. The enclosures were randomized into two treatments, and fed either ice algae or *C. centralis.* Two controls, with ice diatoms and *C. centralis* respectively, were incubated with no copepods. After five days each bottle was sieved through 500 µm mesh and copepods were retained for fatty acid analysis. The bottle content from which copepods had been removed was then filtered (GFF, 25 mm) for chlorophyll *a* (chl *a*) analysis. Samples were extracted in 90% acetone during 24 h at 4°C in the dark. Fluorescence was read on a Turner Designs 10 AU fluorometer before and after acidification, according to Parsons *et al*. [28].

### Fatty acid analysis

Lipids were extracted using two different methods. Lipids from *C. centralis* were extracted using a modified Folch procedure [29] using a 2∶1 chloroform and methanol solution before being transesterified to produce fatty acid methyl esters (FAMEs) using sulfuric acid as a catalyst (Table S1). Ice algae lipids were extracted and transesterified using an *in situ* method similar to Park and Goins [30]. We used a direct method for ice algae instead of the two-step process due to concerns that the limited amounts collected might contain only trace amounts of lipids. The direct method has been found to yield equivalent results when compared to the two-step method and is more efficient for microalgae samples [31]. Duplicate filters were collected for ice algae samples and results were pooled. Individual FAMEs were identified using a gas chromatograph equipped with a flame ionization detector and quantified using 5-α cholestane as an internal standard. FAMEs are referred to in the shorthand A∶Bn-X, where A refers to the number of carbon atoms present, B is the number of double bonds and n-X is the position of the double bond nearest to the methyl terminus.

### Stable isotope analysis

*C. centralis* were freeze-dried at −40°C for 48 h, after lipids were extracted in 2∶1 chloroform and methanol [32], and analyzed for δ^13^C and δ^15^N stable isotopes at the Great Lakes Institute for Environmental Research (University of Windsor, Canada) (Table S1). Ice algae samples were analyzed for δ^13^C and δ^15^N values using a Thermo-Finnigan Delta XP isotope-ratio mass spectrometer (Bremen, Germany) interfaced to an Elemental Analyzer via the Conflo III. Ice algae samples were analyzed at the Stable Isotopes in Nature Laboratory (SINLAB), Canadian Rivers Institute, University of New Brunswick, Canada. Isotopic signatures are expressed as a deviation (δX =  [(**R**sample/**R**standard) −1] ×1,000) from international standards calibrated against Vienna-PeeDee Belemnite (VPDB) for carbon, and atmospheric N_2_ (AIR) for nitrogen.

### Statistical analysis

Statistical analyses were done using R statistical software [33]. Wilcoxon sign rank tests were used to compare PUFA proportions of ice algae and diatoms. Fatty acid composition data were transformed using arcsin square-root functions as appropriate before using parametric multivariate analyses [34]. We used a two-sample Hotelling's T test to compare multivariate fatty acid compositional means of ice algae and *C. centralis* based on 13 fatty acids (R package: rrcov [35]). These 13 fatty acids were the most abundant in samples (>1%) and comprised 90% of total *C. centralis* fatty acids and 91% of ice algal fatty acids. Principal component analysis (PCA; R package: vegan [36]) and hierarchical clustering analysis based on principal components (HCPC; R package: FactoMineR [37]) were used to show differences in fatty acid profiles and internal groupings within *Coscinodiscus* and samples; clustering of ice algae fatty acids were not taken into consideration for this study. Significance of PCA axes were calculated based on Equiprobability  =  (1/#var)* 100 = 1/16*100 = 6.25% [38]. Redundancy analysis of untransformed fatty acid data was used to test the effect of environmental conditions on the fatty acid signature of *C. centralis* [36]. A two-sample t-test assuming equal variance was used to evaluate difference in δ^15^N between clusters. Regressions were used to assess relationships between stable isotopes, environmental variables and lipid abundances. Unless otherwise noted, all fatty acids and stable isotope values are reported as mean ± one standard deviation.

## Results

### Field observations

Mean ice thickness in 2011 was 133.0±19.5 cm (range  = 96.2 to 167.6 cm). Near the end of the study there was open water in Barrow Strait east of Cornwallis Island and several polynyas were present in the northern portion of Wellington Channel. In 2012, the ice was thicker, ranging from 121.4 to 235.0 cm (mean 163±35 cm), and there were no signs of melting or open water in our study area. Mean snow depth at the stations was higher in 2011 (8.3±4.3 cm, range  = 1.1 to 20.6 cm) compared to 2012 (6.1±2.8 cm, range  = 1.7 to 13.9 cm). Downwelling PAR was similar both years, mean  = 1010.0 ±285.3 µE m^−2^ s^−1^ in 2011 and 1056.1±181.9 µE m^−2^ s^−1^ in 2012 while under-ice PAR measured at the stations was lower and less variable in 2011 (mean  = 4.90±3.03 µE m^−2^ s^−1^) than in 2012 (mean  = 9.36±8.76 µE m^−2^ s^−1^). Pennate diatoms dominated the species composition of the sea ice community with *Nitzschia frigida* being the most abundant.

### Stable isotopes and fatty acids in *C. centralis* and ice algae

**Figure 2:**
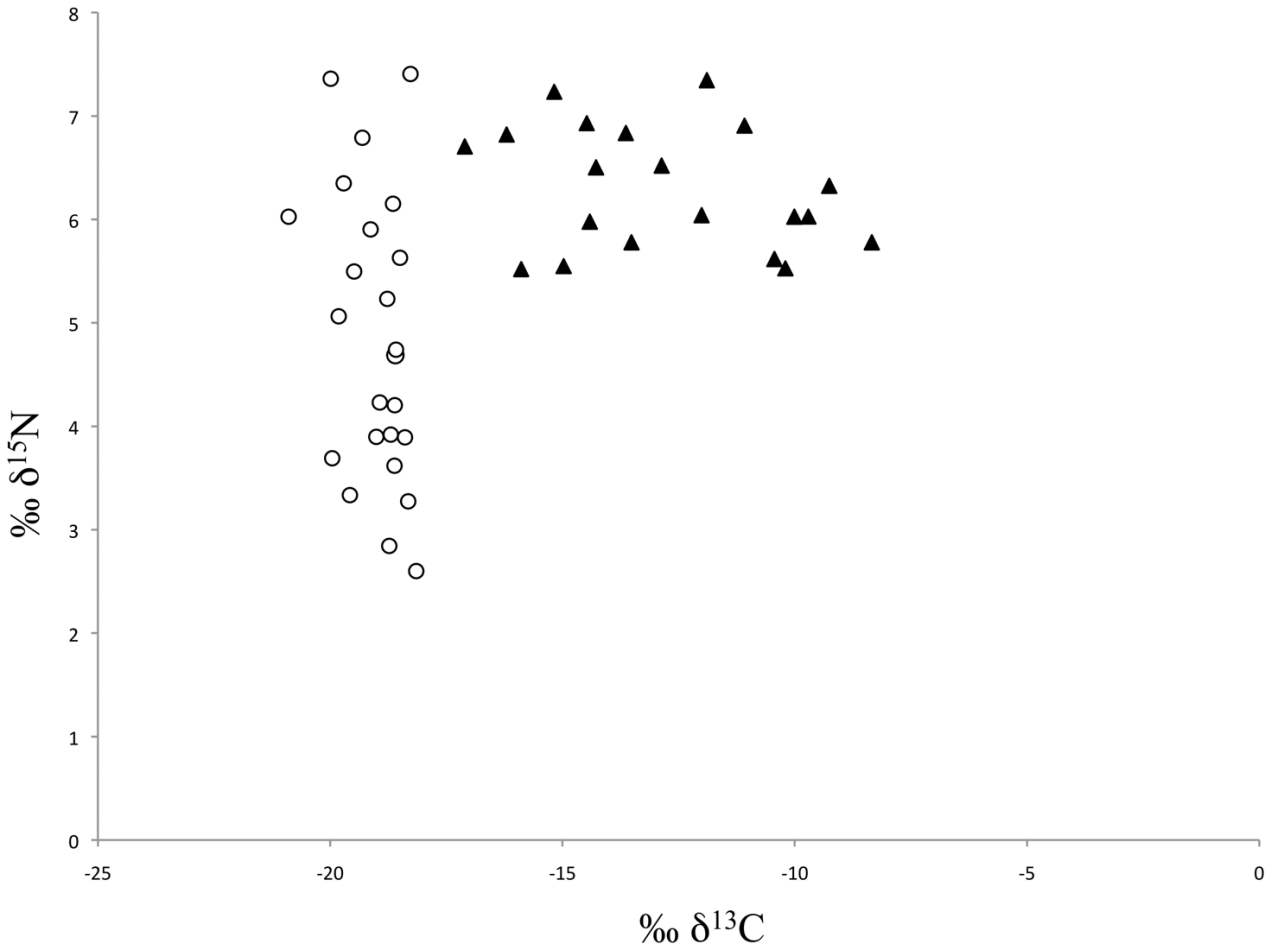
Stable carbon and nitrogen isotope values for *C. centralis* (circles) and ice algae (triangles).

The δ^15^N isotopic signatures for *C. centralis* showed a broader range of values (Fig. 2; 4.95±1.39‰; range: 2.84 to 7.42‰) in comparison to those of ice algae. The latter were significantly more enriched and had more consistent δ^15^N signatures (6.30±0.59‰; t-test, p<0.001). *C. centralis* δ^13^C values were significantly more depleted and less variable (−19.07±0.67‰; t-test, p<0.001) relative to ice-associated δ^13^C values (Fig. 2; −12.77±2.57‰).

**Figure 3:**
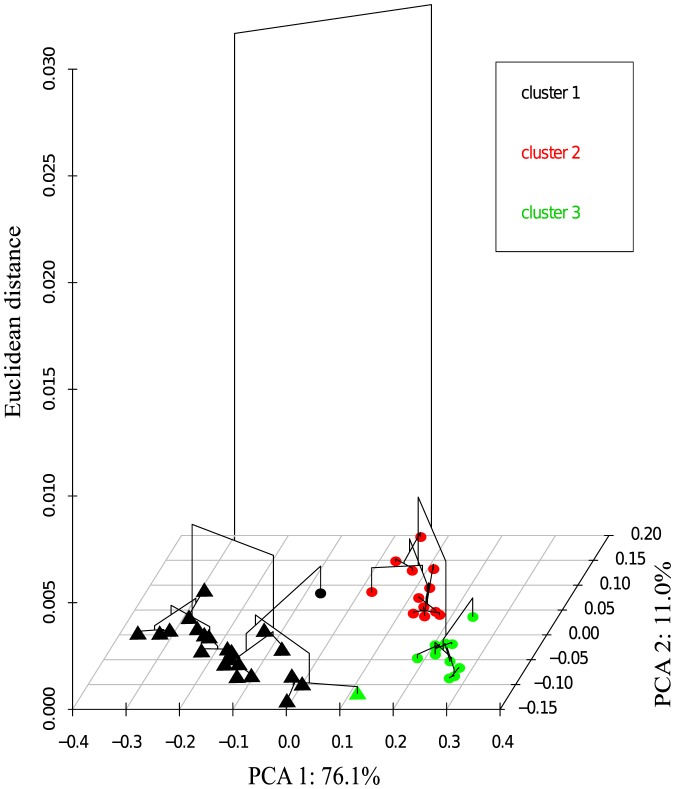
Hierarchical clusters of ice algae (triangles) and *C. centralis* (circles) fatty acid profiles overlaid on the first two principal component axes.

Ice algae and *C. centralis* had significantly different fatty acid compositions (Hotelling's t-test, p<0.001). Pelagic samples had significantly higher abundances of both EPA (Wilcoxon rank sum, p<0.001) and total PUFA (Wilcoxon rank sum, p<0.001) than ice algal communities (Table 1). Ice algae were richer in monounsaturated fatty acids (41.4±7.0% *versus* 26.4±6.3%) whereas *C. centralis* had higher levels of all 18-carbon fatty acids with the exception of 18∶3n-6 (Table 1). Ice algae and pelagic *C. centralis* had similar levels of saturated fatty acids. The environmental conditions at each station had no significant effect on the fatty acid signatures of *C. centralis.* HCPC analysis identified three clusters of samples based on fatty acid profiles. Although the differences in fatty acid composition were largest between ice algae and *C. centralis,* the differences between the two clusters of *C. centralis* were also significant (Fig. 3; MANOVA: Wilks' λ<0.001, p<0.001).

**Table 1:** Mean abundance (±SD) of selected fatty acids (expressed as mass % of total fatty acids) in ice algae and pelagic diatom *Coscinodiscus centralis* collected in spring 2012.

Fatty acid	Ice algae		*C. centralis*	
14∶0*	8.94	±1.97	10.23	±2.21
16∶0*	16.85	±3.57	9.00	±2.67
16∶1n−9	2.81	±2.76	0.12	±0.13
16∶1n−7*	35.21	±7.86	17.41	±5.16
16∶2n−4*	2.91	±0.87	4.79	±1.32
16∶3n−4*	1.42	±0.98	2.78	±1.03
16∶4n−1*	4.68	±2.24	7.13	±2.59
18∶0*	0.36	±0.16	2.01	±1.25
18∶1n−9*	0.63	±0.44	2.35	±1.42
18∶2n−6*	0.47	±0.11	3.53	±1.18
18∶3n−6*	1.22	±0.45	0.53	±0.15
18∶3n−3	0.34	±0.16	0.27	±0.13
18∶4n−3*	2.08	±0.76	2.69	±0.45
20∶1n−9	0.06	±0.05	0.45	±0.47
20∶4n−6	0.24	±0.10	0.03	±0.06
20∶4n−3	0.40	±0.27	0.34	±0.67
20∶5n−3 (EPA)*	13.66	±5.11	24.41	±5.06
22∶1n−11	0.15	±0.11	0.18	±0.49
22∶1n−9	0.02	±0.03	0.32	±0.33
22:6n−3 *	1.77	±0.64	2.66	±0.93
Total SFA	27.97	±4.43	23.32	±3.99
Total MUFA	41.41	±7.03	26.38	±6.30
Total PUFA	30.62	±10.33	50.30	±8.90

*Thirteen fatty acids used for MANOVA, cluster and principal component analyses

*EPA eicosapentaenoic acid, SFA saturated fatty acids, MUFA monounsaturated fatty acids, PUFA polyunsaturated fatty acids*.

**Figure 4:**
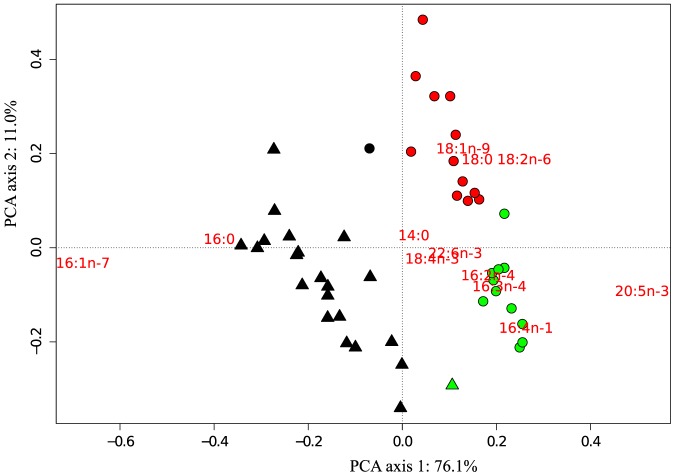
Principal component plot of fatty acid proportions (arcsin square root transformed) of *C. centralis* and ice algae. Colours correspond to the clusters identified in Fig. 4; black indicates cluster 1, red is cluster 2 and green is cluster 3. Fatty acid vectors are scaled proportional to eigenvalues while samples are unscaled.

Equiprobability calculations indicate that only the first two principal component analysis axes were significant, explaining 87.1% of the total variance (Fig. 4). The first axis explained 76.1% of the total variance while the second explained 11.0%. Fatty acids 16∶0, 16∶1n−7, 16∶4n−1, 18∶1n−9, 18∶2n−6, and EPA (20∶5n−3) had the largest eigenvectors in relation to the first two principal component axes (Fig. 4). Separation of the ice algae and *C. centralis* was driven mostly by the presence of 18∶3n−6 in ice algae, and by 18∶2n−6 and 18∶1n−9 in *C. centralis*.

Cluster 2 (red) in the HCPC comprised 12 pelagic samples, with samples in this cluster further from each other in Euclidean distance compared to cluster 3 (green), which contained 11 samples of *C. centralis* and one ice algae sample (Fig. 3). Differences between these two clusters were driven by 18 carbon fatty acids (18∶0, 18∶1n−9, and 18∶2n−6) and 16 carbon PUFA (16∶2n−4, 16∶3n−4 and 16∶4n−1) (Fig. 4). Sites on the eastern portion of McDougall Sound were part of cluster 3 while the western sites grouped in cluster 2. The westernmost station in Resolute Passage was also grouped into cluster 2. The clustering procedure grouped one *C. centralis* sample with the 18 ice algae samples (cluster 1; black), despite the *C. centralis* sample being closer to cluster 2 based on the first two principal component axes (Fig. 3).

**Figure 5:**
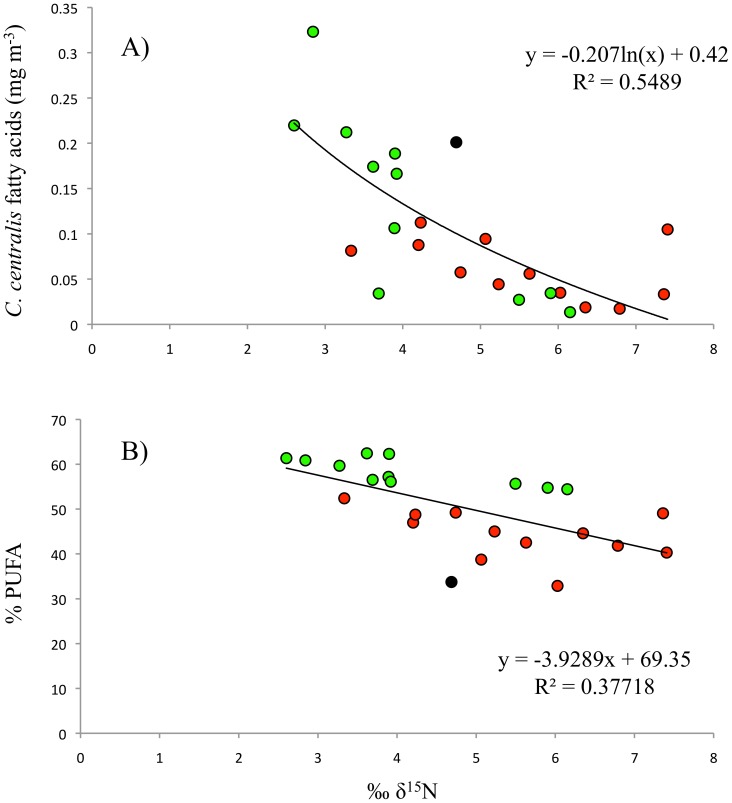
Relationship between δ^15^N and A) concentrations (mg m^−3^) of *C. centralis* fatty acids in the water column and B) PUFA (%) of *C. centralis.* Colours correspond to the clusters identified in Fig. 4; black indicates cluster 1, red is cluster 2 and green is cluster 3.

Fatty acid profiles for *C. centralis* correlated significantly with δ^15^N values. Pelagic samples from Barrow Strait (cluster 3) showed significantly lower δ^15^N values than those from Wellington Channel (cluster 2) (t-test, p = 0.007). Fatty acid concentrations of *C. centralis* were negatively correlated with δ^15^N (Fig. 5A; y = −0.207ln(x)+0.42, r^2^ = 0.55; p <0.001). PUFA concentrations were also negatively correlated with δ^15^N (Fig. 5B; y = −3.9289x+69.35, r^2^ = 0.38 p = 0.001). Three stations in cluster 3 had high values of δ^15^N; two of these were located on the eastern half of the McDougall Sound transect while the other was from the north-west in Wellington Channel (Fig. 5).

### Abundance patterns of *C. centralis*

**Figure 6:**
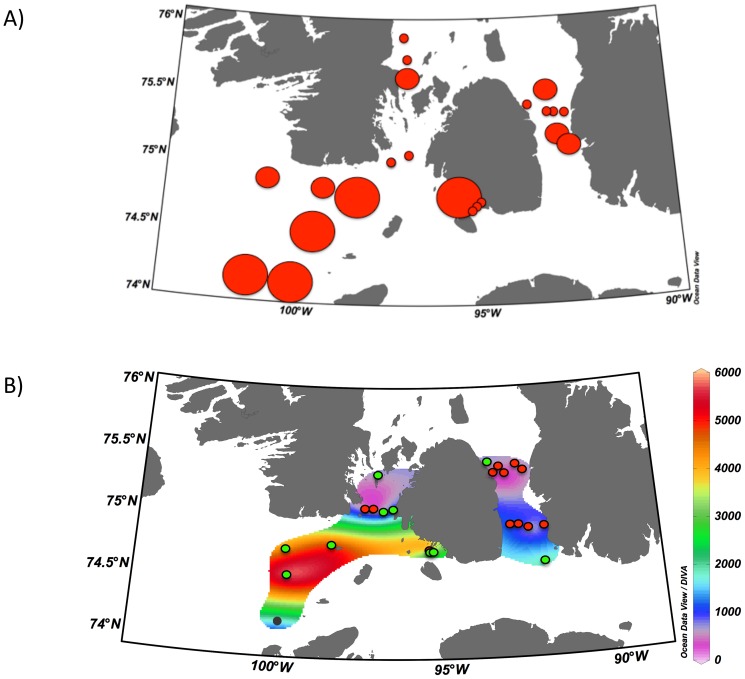
Distribution of *C. centralis* near Cornwallis Island, Nunavut, Canada. **A**) Qualitative estimates of *C. centralis* abundances in spring, 2011. **B**) Densities (cells m^−3^) of *C. centralis* in the water column in spring 2012. Interpolation of cell densities was plotted using Ocean Data View v 4.6.1 [23]. Station colours correspond to the clusters identified in Fig. 4; black indicates cluster 1, red is cluster 2 and green is cluster 3.

Cells of *C. centralis* were found at every station during both sampling years, and accounted, at many stations, for most of the total biomass in net hauls. Total diatom abundances of each station were closely related to cell density (cells m^−3^; r^2^ = 0.81, p<0.001). Qualitative patterns observed in 2011 closely followed the quantitative cell counts obtained in 2012 (Fig. 6) Maximum cell concentration observed was 5713 cells m^−3^ in the western portion of Barrow Strait, while the minimum (201 cells m^−3^) occurred in the north of Wellington Channel (Fig. 6). The size range of *C. centralis* collected in spring 2012 varied from 153 to ∼450 µm in diameter. Almost all pelagic diatoms collected were >250 µm. Cell chloroplasts were clearly visible and contained photosynthetic pigments in all samples observed.

### Feeding experiment

No significant differences were observed between the fatty acid profiles of copepods fed *C. centralis* compared to those fed ice algae. Only two fatty acids changed by more than 2% from initial levels: 16∶0, which increased from 7.7% to 9.8% in copepods fed ice algae (t-test, p = 0.5), and EPA, which decreased in *C. centralis* fed copepods (−3.3±0.8%, p = 0.06). Chlorophyll *a* concentrations were significantly higher in bottles that had both copepods and *C. centralis* than control bottles that contained only *C. centralis* (t-test, p = 0.03). Conversely, there was a no significant difference in chl *a* concentration for ice algae replicates that were incubated with copepods relative to copepod-free controls (t-test, p = 0.2).

## Discussion

Emerging evidence of substantial under-ice pelagic production has the potential to fundamentally change our understanding of Arctic marine food webs [9], [11]. The discovery of substantial numbers of the pelagic centric diatom *C. centralis* under sea ice in the Canadian High Arctic contributes to this evidence and supports indications that biological productivity in this region may be underestimated [39]. Our results suggest that *C. centralis* may be initiating growth both in polynyas and under pack ice and may represent a source of high quality omega-3 fatty acids for Arctic food webs. Despite this, the large cell sizes observed may preclude the consumption of *C. centralis* by herbivorous pelagic zooplankton, specifically calanoid copepods.

*C. centralis* had significantly higher levels of PUFA, specifically the essential fatty acid EPA, compared to ice algae. Although PUFA levels in *C. centralis* varied, presumably depending on the origin of the cells (32–62%) (Fig. 5.B), PUFA were still in the upper ranges of phytoplankton values reported from previous Arctic, cold-water, and laboratory studies [40]–[42]. PUFA levels greater than 30% are indicative of the exponential growth phase of a bloom, where fatty acid deposition is highest in the polar lipids of cell membranes [40], [42]. The similarities in fatty acid profiles and the similar dominance of the characteristic diatom fatty acids EPA and 16∶4n−1 in *C. centralis* during this study and spring blooms in Arctic open water environments [40] strongly suggest that *C. centralis* were actively growing during our study.

The δ^13^C values for *C. centralis* samples were more depleted and less variable than those of ice algae, as expected due to the higher levels of dissolved inorganic carbon available in the water column compared to the ice/water interface during growth (Fig. 2) [43]. As δ^13^C is only slightly enriched with each successive trophic level [44], this may be useful to discern the relative importance of *C. centralis* as a carbon source in Arctic marine food webs. Conversely, ice algae showed relatively small variations in δ^15^N values compared to *C. centralis* (Fig. 2), indicating more stable nitrate accessibility at the ice/water interface than throughout the water column [45].

During the two years of our study, cells of *C. centralis* were found under the ice at every station. Abundance estimates were comparable in 2011 and 2012 indicating that patterns of cell abundance may be consistent between years (Fig. 6). The close relationship between total abundance and cell concentrations (cells m^−3^) indicates that *C. centralis* cells were uniformly distributed throughout the water column. Barrow Strait (cluster 3), an area of mixed ice cover and moving pack ice, showed higher concentrations of fatty acids (0.13±0.1 mg m^−3^; maximum 0.32 mg m^−3^) and cell abundances than McDougall Sound and Wellington Channel (cluster 2) (0.06±0.03 mg m^−3^; maximum 0.11 mg m^−3^) (Fig. 6). Based on the general circulation patterns in this region, the origin of the cells in cluster 3 would be to the west, in the region of Viscount Melville Sound, an area that was covered by pack ice during both years. The second source of *C. centralis* cells (cluster 2) would therefore be located north of Cornwallis Island and had much lower associated abundances.

Inferences on the origin of *C. centralis* cells can be made based on the combination of fatty acid and stable isotope values. In both years under-ice PAR levels were high enough to support photosynthesis (2–9 µE m^−2^ s^−1^) [46]. Phytoplankton PUFA proportions decrease with increasing light levels [40], [47] and to some extent, nutrient limitation [45], [48]. Higher isotopic values in primary producers are indicative of nutrient depletion due the preferential incorporation of lighter isotopes [49], [50]. The significantly lower values for δ^15^N and high PUFA (Fig. 5.B) suggest therefore that *C. centralis* (cluster 3) advected from Viscount Melville Sound had high nitrogen availability and low light conditions. Furthermore, the similarity between individuals within the cluster indicates that conditions were relatively consistent during growth (Fig. 3). In contrast to lower latitudes, the photosynthetic capability of Arctic phytoplankton increases significantly with nitrogen availability [51], which would explain potential growth under very low light conditions below pack ice. Conversely, cells belonging to cluster 2 (Wellington Channel/MacDougall Sound) had significantly higher values of δ^15^N than cluster 3 and lower PUFA abundances (Fig. 5.B). This is consistent with a more depleted nutrient pool and higher light levels, suggesting an open water growth source such as the polynyas of Penny Strait or Dundas Island.

*C. centralis* has previously been reported in the Canadian High Arctic at the time of our study, i.e. in May, in the North Water Polynya [18]. In this region, *C. centralis* was present early during the spring bloom, which was initiated by the combination of sufficient light availability and nutrients to support high photosynthetic activity. Accounts of *Coscinodiscus* occurring as far west as Cornwallis Island are rare, with none indicating the presence of either this particular species, nor the abundance found in this study [52], [53]. Comprehensive spring sampling campaigns from 1980 to the 1990s have no record of *C. centralis*, despite using almost identical water column sampling methods and locations as the current study [10]. However, *Coscinodiscus* are often present in low abundances relative to dominant taxa, and may be missed in quantitative phytoplankton sampling routines [12].

The very large size (250 µm to ∼450 µm in diameter) of *C. centralis* generally observed during our study was greater than the average size range previously reported (180–200 µm, [12], [16]). These larger sizes may be due to the inherent variability of *C. centralis*, which has been found to reach up to 300–372 µm in diameter [12], [13]. Whereas increasing temperatures were found to reduce cell size in marine pelagic diatoms [54], *Coscinodiscus* collections used as the basis for taxonomic identification were all collected from water at or above 3°C [12], [14]. Cold-adapted ecotypes, which are found in other cosmopolitan species of phytoplankton, may explain the larger size of *C. centralis* observed in our study area compared to documented mean cell sizes for this species [55].

The large cell size of *C. centralis* could preclude nauplii or juvenile copepods from effectively grazing on these cells, which may explain the lack of any observable grazing in our feeding experiment. Despite *C. centralis* and ice algae having significantly different fatty acid profiles, no difference was observable in copepods fed either of the treatments. Based on turnover rates of lipids in calanoids, a dietary switch should have been evident via modification in fatty acids within the duration of the experiment if grazing of *C. centralis* did take place [56]. The decreases in copepod EPA were consistent with starvation [57]. Not only did *C. centralis* cells still appear to be healthy at the end of the experiment but chl *a* concentrations significantly increased in bottles containing zooplankton. As irradiance levels were between 2.5 and 3.5 µE m^−2^ s^−1^ during the incubation, this provides a direct observation of shade adaptation in *C. centralis* collected under the ice, a condition necessary for a life history strategy alternate to spring bloom conditions [21].

Our results showed that healthy *C. centralis* cells were present in the Canadian High Arctic under sea ice concurrently with the ice algal bloom, and therefore spring primary production is likely being underestimated in this region. Fatty acid and stable isotope signatures indicated that growth was likely occurring primarily in polynyas and under moving pack ice where light conditions are more favorable than under landfast ice. Given the ability of *C. centralis* to grow under a variety of conditions [12], [21], its ongoing and regular presence at similar or higher latitudes [18], [19] and the lack of significant environmental variation from the region's normal conditions [10], [25], it is probable that *C. centralis* occurs regularly in early spring in this area and may have been overlooked in the past. The presence of *C. centralis* under sea ice represents a source of high quality PUFA during a period when most other pelagic producers are limited; however it is not clear if they can be effectively grazed by copepods. Our results support growing evidence indicating that pelagic primary production may play a biologically important role during periods of ice cover and highlight the need for further investigation of under-ice processes and their impacts on Arctic marine food webs.

## Supporting Information

Table S1 *C. centralis* cell abundances, sampling locations, stable isotope values (δ^15^N and δ^13^C) and fatty acids (expressed as mass % of total fatty acids).
(XLSX)
