# Correction: Large, Omega-3 Rich, Pelagic Diatoms under Arctic Sea Ice: Sources and Implications for Food Webs

**DOI:** 10.1371/journal.pone.0118291

**Published:** 2015-03-05

**Authors:** The PLOS ONE Staff

There is an error in the affiliation of author Gregory W. Thiemann. The affiliation should be: Faculty of Environmental Studies, York University, Toronto, Ontario, Canada.
